# Whole-body cryotherapy does not augment adaptations to high-intensity interval training

**DOI:** 10.1038/s41598-019-48518-1

**Published:** 2019-08-19

**Authors:** James R. Broatch, Mathilde Poignard, Christophe Hausswirth, David J. Bishop, François Bieuzen

**Affiliations:** 1 Institute for Health and Sport (IHES), Victoria University, Melbourne, Australia; 2 Department of Physiology, Australian Institute of Sport, Canberra, Australia; 3 French Institute of Sport (INSEP), Research Department, Laboratory Sport, Expertise and Performance, Paris, France; 4 French Tennis Federation, National Tennis Centre, Paris, France; 5 Université Côte d’Azur, LAMHESS, Nice, France; 6 Mouratoglou Tennis Academy, Medical Centre, Biot, France; 7 School of Medical and Health Sciences, Edith Cowan University, Perth, Australia; 8 Institut National du Sport du Québec, Montréal, Canada

**Keywords:** Metabolism, Physiology, Adrenal cortex hormones

## Abstract

The aim of this study was to investigate the effects of regular post-exercise whole-body cryotherapy (WBC) on physiological and performance adaptations to high-intensity interval training (HIT). In a two-group parallel design, twenty-two well-trained males performed four weeks of cycling HIT, with each session immediately followed by 3 min of WBC (−110 °C) or a passive control (CON). To assess the effects of WBC on the adaptive response to HIT, participants performed the following cycling tests before and after the training period; a graded exercise test (GXT), a time-to-exhaustion test (T_max_), a 20-km time trial (20_TT_), and a 120-min submaximal test (SM_120_). Blood samples were taken before and after training to measure changes in basal adrenal hormones (adrenaline, noradrenaline, and cortisol). Sleep patterns were also assessed during training via wrist actigraphy. As compared with CON, the administration of WBC after each training session during four weeks of HIT had no effect on peak oxygen uptake ($\dot{{\rm{V}}}$O_2peak_) and peak aerobic power (P_peak_) achieved during the GXT, T_max_ duration and work performed (W_Tmax_), 20_TT_ performance, substrate oxidation during the SM_120_, basal adrenaline/noradrenaline/cortisol concentrations, or sleep patterns (*P* > 0.05). These findings suggest that regular post-exercise WBC is not an effective strategy to augment training-induced aerobic adaptations to four weeks of HIT.

## Introduction

Whole-body cryotherapy (WBC) consists of short duration (3 to 4 min) exposures to dry, very-cold (−110 °C to −160 °C) air^1^. Traditionally utilized in medicine to relieve pain and inflammatory symptoms associated with chronic pathological conditions (e.g., rheumatic disorders, arthritis, and fibromyalgia), this cryotherapy technique is also used in an athletic context in attempt to improve aspects of exercise performance and recovery^2^. The purported physiological benefits of WBC in an athletic setting have been attributed to cold-induced analgesia, reduced muscle temperature, and suppressed inflammation^3^. Consequently, WBC has been reported to improve endurance exercise performance^4^ and muscle strength^5^, reduce perceptions of pain and fatigue^6,7^, and attenuate markers of exercise-induced muscle damage and inflammation^8^. In addition, WBC has been reported to improve sleep quality^9^, to increase parasympathetic reactivation^1^, and to alter hormonal responses related to pain, stress and inflammation (e.g., catecholamines and cortisol)^10^; these are all parameters that may aid exercise performance and/or recovery from exercise.

Studies investigating the effects of a single WBC exposure on aspects of exercise performance and recovery are mixed, with data reporting beneficial^4,11,12^, negligible^13–15^, and even negative^16^ outcomes. However, considering some (but not all) have reported beneficial effects, a number of studies have investigated if these effects are conserved with repeated WBC exposure during a short-term training period (i.e., 5 to 14 d)^7,9,17,18^. For example, daily WBC administered after intensified training for 14 consecutive days enhanced 400-m swimming time-trial performance in synchronized swimmers, when compared with a passive control^19^. In addition, daily WBC improved performance supercompensation after a simulated one-week taper in functionally overreached endurance athletes^20^. Repeated WBC may also be effective in reducing systemic markers of muscle damage following damaging exercise^7,18^, consistent with that reported following a single exercise session^8^. Research investigating the effects of repeated WBC on immune and inflammatory responses are limited, with regular WBC being reported to increase^7^, decrease^18^, or have no effect^21^ on circulating cortisol concentrations, and dampen the post-exercise inflammatory response^7^. Daily WBC for a period of up to 14 days has also been reported to attenuate exercise-induced increases in ratings of exertion^9^, to improve sleep quality^9^, and to improve antioxidant status^17^. Taken together, these studies demonstrate that regular WBC may be a worthwhile strategy to help maintain exercise performance and recovery status, while concomitantly preventing fatigue accumulation and the exacerbation of muscle damage during short-term training periods^9^.

An important limitation to date is the lack of research investigating the effects of regular WBC on physiological and performance adaptive responses to extended periods of exercise training (i.e., ≥4 wk). This is particularly pertinent considering recent research identifying regular post-exercise cryotherapy, in the form of cold-water immersion (CWI), as a novel method to promote certain alterations towards a more aerobic phenotype^22^. For example, regular post-exercise CWI during ~4 weeks of high-intensity interval running training has been reported to augment the exercise-induced increase in some proteins associated with mitochondrial biogenesis^22^. These alterations were hypothesized to be at least partially mediated by repeated cold-induced *β*-adrenergic activation^22^, a mechanism by which WBC is also hypothesized to assist in the recovery from exercise^1^. Although CWI may be more effective in reducing muscle temperature^23^, WBC has been reported to elicit comparable reductions in tissue and core temperatures as compared with CWI^24^, implicating it as an alternate method by which to potentially promote mitochondrial biogenesis^25^. Regular WBC may also aid the adaptive response to exercise training via improvements in skeletal muscle recovery^3^ and preservation of sleep quantity^9^, thereby improving subsequent training quality. Given the increasing popularity of WBC within training regimes, clarification of its merit in promoting favourable adaptations to exercise training is warranted.

We investigated, for the first time, the effects of regular post-exercise WBC on physiological and performance adaptations to endurance exercise training. It was hypothesized that repeated WBC after exercise training would promote favourable adaptations to a number of parameters implicated in short-term exposures to WBC, including hormonal balance and sleep patterns. Considering the equivocal findings with other forms of regular cryotherapy application^25^, we also investigated whether regular WBC may serve as a novel stimulus to augment training-induced aerobic adaptations, as measured by an improvement in endurance exercise performance.

## Results

### Training

Both groups performed similar volumes of the prescribed HIT over the four weeks (4.96 ± 0.97 MJ and 5.10 ± 1.15 MJ for CON and WBC, respectively; *P* = 0.379). Participants performed 97.0 ± 12.7% of the target volume (work completed) during the entire 4-week training period.

### GXT and T_max_

**Figure 1:**
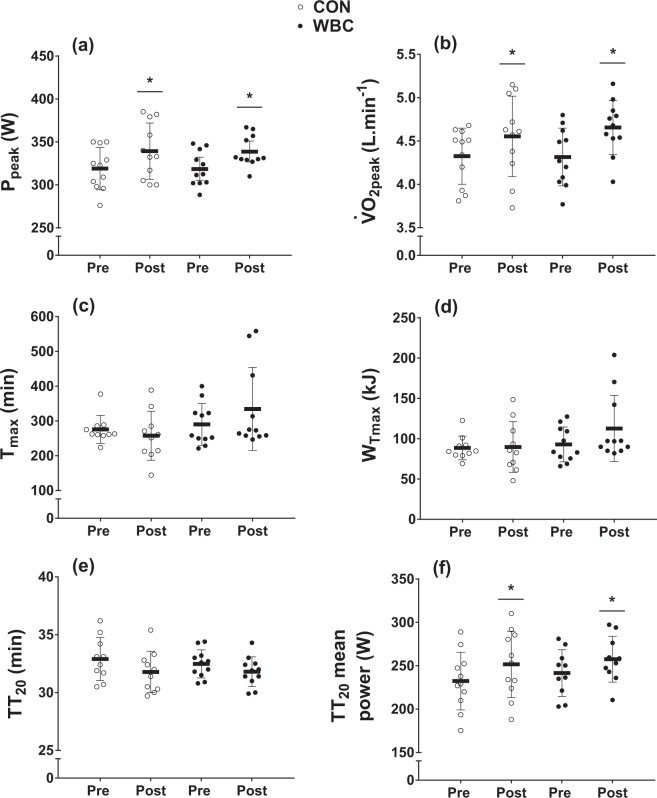
Peak aerobic power (P_peak_; **a**) and peak oxygen uptake ($\dot{{\rm{V}}}$O_2peak_; **b**) achieved during the graded exercise test (GXT), time-to-exhaustion duration (T_max_; **c**), time-to-exhaustion work completed (W_Tmax_; **d**), and 20 km time trial (TT_20_) duration (**e**) and mean power (**f**) for the control (CON, *n* = 11) and whole-body cryotherapy (WBC, *n* = 11) conditions, before (Pre) and after (Post) the 4-week training period. *Significantly higher Post, as compared with Pre. Values are presented as mean ± SD.

There was a main effect of time (*P < *0.001) for P_peak_ (Fig. 1a), which increased by 6.3 ± 3.7% (ES = 0.71 ± 0.23) and 5.9 ± 4.9% (ES = 0.83 ± 0.37) in the CON and WBC groups, respectively. Similarly, there was a main effect of time for $\dot{{\rm{V}}}$O_2peak_ (*P* < 0.001; Fig. 1b), which increased by 5.0 ± 5.6% (ES = 0.34 ± 0.21) and 8.0 ± 7.9% (ES = 0.56 ± 0.30) in the CON and WBC groups, respectively. There were no interaction effects for P_peak_ (*P* = 0.988) or $\dot{{\rm{V}}}$O_2peak_ (*P* = 0.402).

There was no main effect of time nor an interaction effect for T_max_ (*P* = 0.850 and 0.111, respectively), which increased by 3.9 ± 29.0% and 25.6 ± 50.2% (ES = 1.22 ± 1.41, CON *vs*. WBC) in the CON and WBC groups, respectively. Similarly, there was no main effect of time nor an interaction effect for W_Tmax_ (*P* = 0.192 and 0.244, respectively), which increased by 2.7 ± 26.3% and 19.4 ± 50.7% (ES = 1.01 ± 1.20, CON *vs*. WBC) in the CON and WBC groups, respectively (Fig. 1c,d).

### 20-km Time trial (TT_20_)

There was a main effect of time for TT_20_ mean power (*P* = 0.002), which increased by 8.4 ± 7.0% (ES = 0.50 ± 0.23) and 6.9 ± 7.5% (ES = 0.54 ± 0.35) in the CON and WBC groups, respectively. There was no main effect of time for TT_20_ duration (*P* = 0.153), nor interaction effects for either TT_20_ power (*P* = 0.644) or duration (*P* = 0.765) (Fig. 1e,f).

### Sub-maximal test (SM_120_)

**Figure 2:**
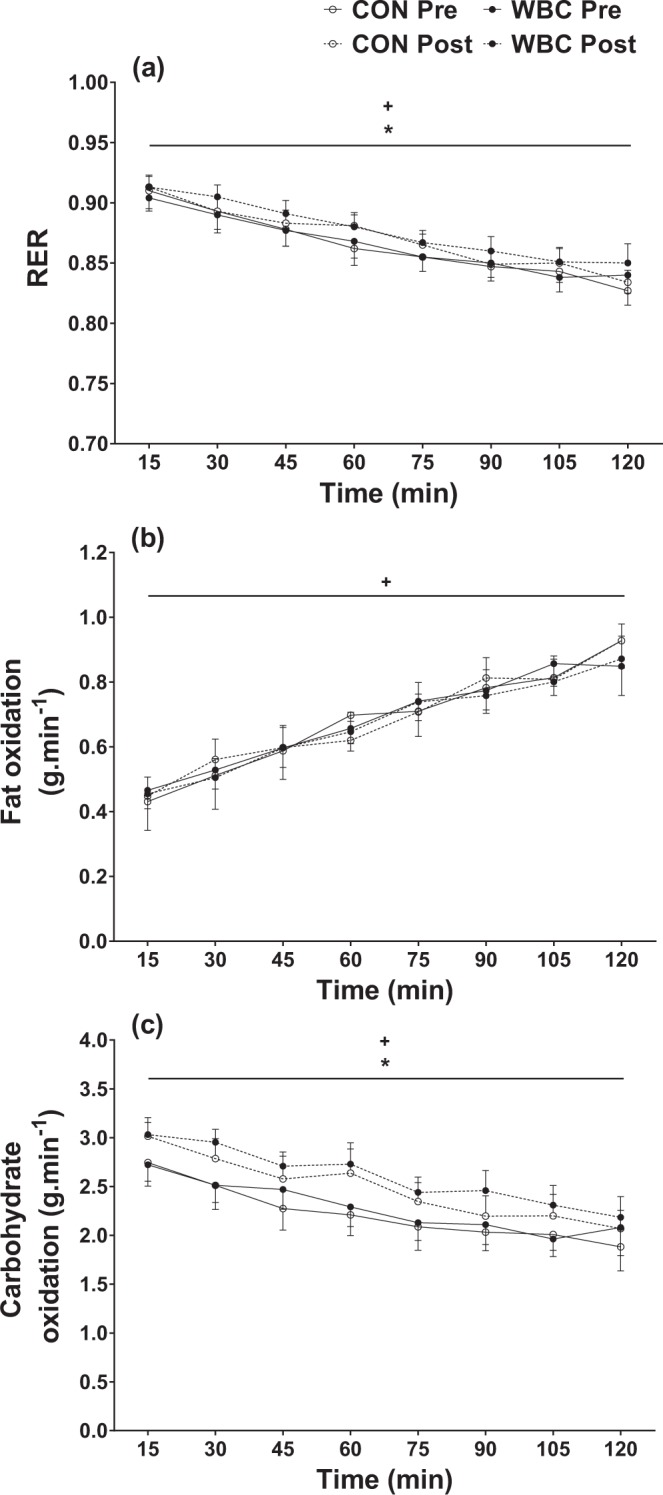
Respiratory exchange ratio (RER) (**a**), fat oxidation (**b**), and carbohydrate oxidation (**c**) during the 120-min submaximal cycling test (SM_120_) for the control (CON, *n* = 11) and whole-body cryotherapy (WBC, *n* = 11) conditions, before (Pre) and after (Post) the 4-week training period. *Significantly higher Post, as compared with Pre; ^+^Significant duration effect during SM_120_. Values are presented as mean ± SD.

There were significant effects of duration and time for RER, with RER progressively decreasing during the SM_120_ (*P < *0.001) and significantly higher post-training (*P* = 0.030). There were no interaction effects for RER during the SM_120_ (*P* = 0.983) or as a result of training (*P* = 0.551) (Fig. 2a). There was a significant effect of duration for fat oxidation rates, which progressively increased during the SM_120_ (*P < *0.001). However, there was no effect of time for fat oxidation rates as a result of training (*P* = 0.845). There were also no interaction effects for fat oxidation rates during the SM_120_ (*P* = 0.980) or as a result of training (*P* = 0.767) (Fig. 2b). There were significant effects of duration for CHO oxidation rates, which progressively decreased during the SM_120_ (*P* < 0.001) and were significantly higher post training (*P* < 0.001). There were no interaction effects for CHO oxidation rates during the SM_120_ (*P* = 0.998) or as a result of training (*P* = 0.684) (Fig. 2c).

### Blood markers

**Figure 3:**
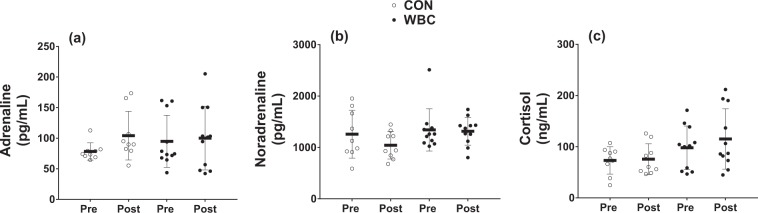
Adrenaline (**a**), noradrenaline (**b**), and cortisol (**c**) concentrations for the control (CON, *n* = 9) and whole-body cryotherapy (WBC, *n* = 11) conditions, before (Pre) and after (Post) the 4-week training period. Values are presented as mean ± SD.

There was no main effect of time nor an interaction effect for basal adrenaline concentration (*P* = 0.296 and 0.180, respectively), which increased by 39.3 ± 68.6% and 25.1 ± 86.2% (ES = 0.69 ± 1.33, CON *vs*. WBC) in the CON and WBC groups, respectively (Fig. 3a). Similarly, there was no main effect of time nor an interaction effect for basal noradrenaline concentration (*P* = 0.455 and 0.655, respectively), which increased by 8.4 ± 34.2% and 2.1 ± 24.9% (ES 0.46 ± 0.87, CON *vs*. WBC) in the CON and WBC condition, respectively (Fig. 3b). In addition, there was no main effect of time nor an interaction effect for basal cortisol concentration (*P* = 0.215 and 0.345, respectively) which increased by 12.1 ± 41.2% and 14.3 ± 24.3% (ES = 0.59 ± 0.77, CON *vs*. WBC) in the CON and WBC groups, respectively (Fig. 3c).

### Sleep

There were no main effects of time for bed time (*P* = 0.797), get up time (*P* = 0.413), time in bed (*P* = 0.184), sleep duration (*P* = 0.131), sleep latency (*P* = 0.325), sleep efficiency (*P* = 0.838), or moving time (*P* = 0.677). There were also no interaction effects for bed time (*P* = 0.988), get up time (*P* = 0.446), time in bed (*P* = 0.558), sleep duration (*P* = 0.367), sleep latency (*P* = 0.325), sleep efficiency (*P* = 0.408), or moving time (*P* = 0.575) (Table 1).

**Table 1:** Changes in sleep variables as a result of four weeks of high-intensity interval training for the control (CON, *n* = 11) and whole-body cryotherapy (WBC, *n* = 11) conditions.

	Bed Time (h:min)	Get up time (h:min)	Time in bed (h:min)	Sleep duration (h:min)	Sleep latency (h:min)	Sleep Efficiency (%)	Moving time (min)
CON	−0:01 ± 0:34	0:19 ± 0:59	0:20 ± 0:45	0:21 ± 0:37	−0:02 ± 0:05	0.84 ± 2.78	-1 ± 3
WBC	−0:01 ± 0:32	0:06 ± 0:27	0:08 ± 0:40	0:05 ± 0:32	0:00 ± 0:05	−0.51 ± 3.59	0 ± 3
**CON vs WBC**							
Difference in means ± 90% CI	0:00 ± 0:28	−0:13 ± 0:39	−0:12 ± 0:26	−0:15 ± 0:30	0:02 ± 0:04	−1.35 ± 2.78	1 ± 3
ES for difference ± 90% CI	0.00 ± 0.66	−0.11 ± 0.72	−0.21 ± 0.72	−0.32 ± 0.66	0.70 ± 1.09	−0.29 ± 0.59	0.17 ± 0.59

For training-induced changes, negative results represent a decrease, whereas positive results represent an increase in the measures reported. CI, confidence intervals. ES, effect size. Measurements, mean ± SD.

## Discussion

The main finding of this study was that four weeks of HIT improved peak aerobic power, $\dot{{\rm{V}}}$O_2peak_, and cycling time-trial performance, and also increased carbohydrate oxidation rates and RER. However, regular WBC performed after each training session had no effect on training-induced changes in these parameters.

Consistent with previous research^26^, the results of the current study support that HIT performed at intensities of P_peak_ and durations of 60% of T_max_ is an effective means to increase P_peak_, $\dot{{\rm{V}}}$O_2peak_, and time-trial performance in already well-trained cyclists. Although the fitness of the participants in the current study was less than cyclists previously recruited by Laursen *et al*.^26^ (P_peak_, 319 *vs*. 439 W; $\dot{{\rm{V}}}$O_2peak_, 59.6 *vs*. 66.5 mL.kg^−1^.min^−1^; W_Tmax_, 91 *vs*. 121 kJ), training-induced increases in P_peak_ (6.1 *vs*. 4.7%), absolute $\dot{{\rm{V}}}$O_2peak_ (6.5 *vs*. 5.4%), and endurance performance (7.5 *vs*. 5.2%) were of similar magnitudes. As previously described, performance improvements as a result of incorporating HIT into the already high training volume of the well-trained athlete are likely to have occurred in parallel with a number of physiological adaptations, including improvements in muscle buffer capacity^27^, the ventilatory threshold^28^, the lactate threshold^29^, and motor unit recruitment^30^.

An important aspect of this research was to determine for the first time whether regular WBC had any effect on training-induced gains in cycling performance. Despite increases in P_peak_ and TT_20_ mean power, regular post-exercise WBC during 4 weeks of HIT had no significant effect on improvements in these markers of endurance performance, nor T_max_ or W_Tmax_. This is consistent with the reported effects of regular CWI on cycling performance^31,32^, and thus supports the notion that regular cryotherapy during an endurance exercise training period has limited effect on augmenting training-induced changes in aerobic adaptations^25^. In the current study, the large (but not significant) differences between conditions following training for T_max_ (3.9% for CON *vs*. 25.6% for WBC) and W_Tmax_ (2.7% for CON *vs*. 19.4% for WBC), as well as the larger inter-subject variability and potential type II error, suggests that further research is warranted to investigate the effects of WBC on exercise performance. In particular, further research is required to determine if the large improvements for some participants in the WBC group is related to random measurement error or suggests that some individuals are more responsive to WBC following training; i.e., some individuals may have a more pronounced response to cold stimulation (e.g., thermogenesis) than others, and therefore a differing molecular and adaptive response to post-exercise WBC^25^.

Despite reports that repeated WBC can improve or maintain endurance exercise performance in the short-term^19,20^, this study provides evidence that regular WBC during 4 weeks of cycling HIT has limited influence on performance markers of endurance adaptations. A potential explanation for these discrepancies is that in these studies WBC was used daily as a method to limit the signs of overreaching^9^ or to speed up supercompensation during a taper in functionally overreached athletes^20^. Absence of a WBC-induced effect on exercise performance in the current study is also supported by the lack of a significant difference between conditions for $\dot{{\rm{V}}}$O_2peak_, substrate utilization, or catecholamine concentrations (discussed below). Furthermore, these data support previously-published research investigating the effects of regular cold-water immersion following cycling training, whereby CWI performed regularly during 3 to 4 weeks of cycle training had no effect of performance adaptations in competitive cyclists^33^ or recreationally-active males^31^.

To understand the mechanisms surrounding the possible effects of WBC, humoral responses related to stress and inflammation are commonly investigated^10^. In the current study, we investigated the effects of regular post-exercise WBC during 4 weeks of high-intensity cycling on the basal concentrations of the adrenal hormones cortisol, adrenaline, and noradrenaline. Consistent with previous research demonstrating no change in the response to endurance training^34^, basal cortisol levels were unchanged in the control group; this may reflect a lack of long-term training stress and overtraining^35^. Similarly, the additional stress imposed by repeated WBC also had no effect on training-induced changes in cortisol (Fig. 3c). This is not entirely unexpected considering the administration of repeated WBC has been reported to increase^7^, have no effect^21^, or decrease^18^ basal cortisol levels, highlighting the equivocal nature of research performed to date. A potential explanation for these contrasting findings is the varying WBC (e.g., daily post-exercise *vs*. twice daily pre- and post-exercise) and/or exercise (e.g., cycling *vs*. multi-disciplinary training) protocols used across studies, as it can be assumed that different training forms^36^ and levels of cold exposure^37^ also lead to different hormonal adaptations.

In the current study, basal adrenaline and noradrenaline concentrations were unchanged following training in both the CON and WBC group. Although basal concentrations of these catecholamines may be elevated in endurance^38^ and sprint-trained^39^ individuals as compared with untrained individuals, these concentrations typically do not change following a period of regular training^40^. In fact, it has been suggested that the training-induced changes observed in previous studies may be explained by the individual’s emotive feelings^39^. In the only study to date to investigate adrenaline and noradrenaline levels following repeated WBC, Leppaluoto *et al*.^10^ reported no change in adrenaline, but a significant increase in basal noradrenaline, following 12 weeks of passive WBC. A potential explanation for the inconsistencies in noradrenaline changes between the current study and Leppaluoto *et al*.^10^ is the preceding exercise stimulus. Considering long-term catecholamine adaptations to exercise are still for the most part unclear^41^, hormonal responses to passive or post-exercise WBC may be vastly different. Although the current study provides new data regarding the response of adrenal hormones with regular post-exercise WBC, further work to clarify their response is warranted.

A novel component of this study was to investigate the effects of regular post-exercise WBC on physiological markers of aerobic training adaptations, including $\dot{{\rm{V}}}$O_2peak_ and substrate utilization. Ten to 15 min of cold exposure following endurance exercise has previously been implicated in the activation of signalling pathways associated with mitochondrial biogenesis^42^, responses which may be largely related to cold-induced thermogenesis. However, $\dot{{\rm{V}}}$O_2peak_ was unaltered following regular post-exercise WBC, consistent with previous research utilizing regular cold-water immersion (~10 °C) as a post-exercise cryotherapy intervention^31^. This is not surprising however, as $\dot{{\rm{V}}}$O_2peak_ is mostly determined by central adaptations (e.g., cardiac output)^43^, whereas WBC is more likely to affect peripheral adaptations (e.g., oxygen supply to the muscle, lipid metabolism, etc.)^44^. Furthermore, a potential explanation for the lack of effect of regular WBC on $\dot{{\rm{V}}}$O_2peak_ is that the level of cold stress administered during a 3-min WBC exposure is not a large enough stress to alter exercise-induced training adaptations. For example, considering the previously reported increases in mitochondrial proteins in animals exposed to cold air for 24 h per day^45^, a larger cold-stress (i.e., colder and/or longer) may be needed to elicit significant alterations in aerobic-related training adaptations in humans^25^.

We also hypothesized that the intense cold stimulus induced by regular WBC would alter thermogenesis and lipid metabolism, consistent with alterations in the cholesterol profile previously reported following WBC^8^. It is well-established that the percentage of total energy derived from fat and carbohydrates progressively increases and decreases (respectively) as a result of prolonged aerobic training^46,47^. However, this was not supported in the current study as fat and carbohydrate rates, as measured by indirect calorimetry, were respectively unchanged or increased in both groups during the SM_120_. A likely explanation for these data is that the SM_120_ exercise intensity was prescribed relative to the pre- or post-training GXT (60% P_peak_), meaning the post-training SM_120_ workload was 6.1% higher compared with the pre-training workload. As such, the observed training-induced increases in carbohydrate oxidation and RER, as well as no change in fat oxidation, are likely the result of an increased workload. When comparing between conditions, there was no significant effect of regular post-exercise WBC, consistent with previous observations^7,8^. Consistent with previously suggestions^25^, a larger cold-stress may have been needed to elicit significant alterations in substrate utilisation.

Only one study to date has investigated the effects of regular WBC during an intensified training period on sleep patterns. Schaal *et al*.^9^ administered daily WBC during a 14-day intensified training program in synchronized swimmers and reported improvements in sleep duration and sleep latency compared with a passive control. The authors suggested that these WBC-induced improvements in sleep quantity might be mediated by a cold-induced increase in noradrenaline concentrations^1^ and a resultant improvement in parasympathetic reactivation^19^. In the current study, regular post-exercise WBC had no effect on markers of sleep quantity. Noradrenaline concentrations were also unchanged between conditions, consistent with the hypothesis that WBC may improve parasympathetic reactivation and sleep quantity via an increase in circulatory noradrenaline levels^9^.

The most important findings from the current study were that regular post-exercise WBC administered during a 4-week high-intensity interval cycling training period had no advantageous effect on endurance-related training adaptations and cycling performance, basal catecholamine/cortisol concentrations, or sleep quality. As such, these findings suggest that regular WBC is not an effective strategy to augment training-induced aerobic adaptations following HIT. However, it is important to note that regular WBC also had no detrimental effect on physiological and performance adaptations related to HIT. Moreover, regular WBC may potentially have advantageous impacts elsewhere (e.g., exercise-induced muscle damage, placebo effect etc.), and as such there is no reason to avoid its administration during athletic training regimes.

## Methods

### Participants

Twenty-two healthy males completed this study (Table 2). Written informed consent was obtained prior to participation, and all participants were screened for cardiovascular risk factors associated with exercise and WBC. All participants were recreational athletes (triathlon or cycling), who trained a minimum 6 h/wk and were unaccustomed to WBC. The study employed a two-group, parallel-group design, in which participants were assigned to one of two conditions in a randomized, counter-balanced fashion (detailed below). Due to a change in availability for testing and/or training sessions, two additional participants began testing but dropped out during the training intervention (one participant from each condition). All procedures and methods were approved by the Institution’s Human Research Ethics Committee (CPP Île de France 8 – ref. 2015-A00561-48) and performed in accordance with the relevant guidelines and regulations.

**Table 2:** Participant characteristics.

Variable	CON Group (*n* = 11)	WBC Group (*n* = 11)
Age (years)	37 ± 9	37 ± 8
Height (cm)	178.0 ± 9.1	180.6 ± 9.1
Body mass (kg)	73.2 ± 8.5	73.4 ± 8.2
Body mass index (kg/m^2^)	23.1 ± 2.6	22.5 ± 1.7
$\dot{{\rm{V}}}$O_2peak_ (mL·kg^−1^·min^−1^)	59.8 ± 7.6	59.4 ± 7.3
P_peak_ (Watts)	319.0 ± 24.7	319.0 ± 20.2

Values are means ± SD. CON, control; WBC, whole-body cryotherapy; $\dot{{\rm{V}}}$O_2peak_, peak oxygen uptake; P_peak_, peak aerobic power; There were no differences between groups for any of the descriptive characteristic.

### Experimental overview

Prior to familiarisation and baseline testing, participants attended a preliminary medical consultation with a doctor employed at the institution. During this consultation participants were screened for any contraindications for WBC and were provided medical clearance to complete the study. Following this consultation, participants reported to the laboratory for a familiarisation of the equipment and testing procedures, including exposure to WBC.

**Figure 4:**
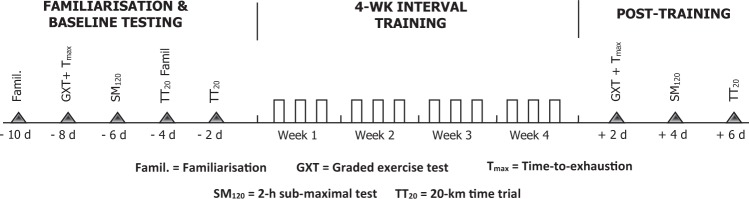
Experimental Design.

Briefly, the experimental protocol consisted of i) familiarisation and baseline testing; ii) a 4-week interval training intervention with or without WBC, and iii) post-training testing (Fig. 4). For baseline testing, participants attended the laboratory on four separate occasions to complete a graded exercise test (GXT) and time-to-exhaustion test (T_max_), 120-min sub-maximal cycling test (SM_120_), and two (familiarization and experimental) 20-km cycling time trials (TT_20_). Participants then completed twelve sessions of high-intensity interval training (HIT) over a period of four weeks, with each training session followed immediately (~15 min) by their assigned condition (WBC or seated rest). Approximately 48 h after the final training session, participants completed post-training GXT, T_max_, SM_120_, and TT_20_ trials. The timing and nature of post-training testing was identical to baseline testing, and all experimental trials were separated by 48 h.

Participants were instructed to abstain from alcohol 24 h prior and food 2 h prior to each of the baseline and post-training trial days. Food diaries were recorded for the 24 h preceding each baseline testing session, and participants were asked to replicate this diet for the 24 h preceding the corresponding post-training testing session. Participants were asked to maintain their regular level of training throughout the study, and training volume/mode/intensity was collected via a web-based training diary (Garmin Connect, USA). Following the pre-training tests, participants were pair-matched according to peak oxygen uptake ($\dot{{\rm{V}}}$O_2peak_) achieved during the GXT. Pairs were then allocated to one of the two training groups. When all allocations were complete, the training groups were randomly allocated to either a passive control (CON) or WBC condition. To limit the effects of circadian rhythm on the dependent measures, matched pairs performed all training and testing sessions at the same time of day.

### Graded exercise test (GXT)

Participants performed a continuous GXT on an electronically-braked cycle ergometer (Lode, Groningen, The Netherlands) to determine their peak oxygen uptake ($\dot{{\rm{V}}}$O_2peak_) and peak aerobic power (P_peak_). Following a 3-min steady state warm-up at 100 W, the workload was increased by 25 W every 2 min until the participant reached volitional fatigue. Participants were instructed to maintain a pedalling cadence of between 80 and 90 rpm and wore a heart rate monitor (Cosmed Wireless HR Monitor, Rome, Italy) during the test. The test was stopped when pedalling cadence dropped below 60 rpm. During the entire test, participants wore a mask covering the nose and mouth (Hans Rudolph, Kansas City, USA), and breath-by-breath expired gases were analysed by a metabolic cart (Quark CPET, Cosmed, Rome, Italy) calibrated to known gas concentrations. Expired $\dot{{\rm{V}}}$O_2_ values were averaged over 15-s periods, and $\dot{{\rm{V}}}$O_2peak_ was defined as the average of the two highest consecutive values (L.min^−1^) reached during the test. P_peak_ was calculated as follows: ${{\rm{P}}}_{{\rm{peak}}}={{\rm{P}}}_{{\rm{final}}}+(\frac{t}{120}\cdot 25)$; where P_final_ was the power output of the last completed stage, and *t* was the time in seconds of any final uncompleted stage (stage duration and workload increment were 120 s and 25 W, respectively).

### Time to exhaustion test (T_max_)

Exactly 1 h after volitional fatigue in the GXT, participants performed a time-to-exhaustion test (T_max_). This test was comprised of a 5-min warm-up at 100 W, followed immediately by a steady-state cycle to fatigue at the P_peak_ achieved during the preceding GXT. Participants were instructed to maintain a cadence of between 90 and 100 rpm, and the test was stopped when pedalling cadence dropped below 60 rpm. Work completed during T_max_ (W_Tmax_) was calculated as the product of P_peak_ and T_max_^26^_._

### Submaximal test (SM_120_)

The SM_120_ was performed pre and post HIT to assess the effects of regular WBC on substrate utilization rates. Following a 5-min warm-up at 100 W, participants performed the SM_120_ at a pedalling cadence above 60 rpm. Participants cycled for 120 min at a workload of 60% P_peak_, calculated according to the corresponding P_peak_ achieved during the pre- or post-training GXT. Expired air samples were collected during the 5-min periods preceding each 15-min interval (i.e., 15, 30, 45, 60, 75, 90, 105 and 120 min) of the exercise bout. Collection and analyses of expired gases were performed as described in the GXT protocol. Values of oxygen consumption ($\dot{{\rm{V}}}$O_2_) and carbon dioxide production ($\dot{{\rm{V}}}$CO_2_) were averaged during the last 3 min of each sampling period to calculate rates of whole-body fat and carbohydrate oxidation based on the following non-protein respiratory quotient formulas^48^

$$\begin{array}{c}{\rm{Carbohydrate}}\,{\rm{oxidation}}=4.585{\dot{{\rm{V}}}{\rm{O}}}_{{\rm{2}}}-3.226{\dot{{\rm{V}}}\mathrm{CO}}_{{\rm{2}}}\\ \,{\rm{Fat}}\,{\rm{oxidation}}={\rm{1}}{\mathrm{.695}\dot{{\rm{V}}}{\rm{O}}}_{{\rm{2}}}-{\rm{1}}{\mathrm{.701}\dot{{\rm{V}}}\mathrm{CO}}_{{\rm{2}}}\end{array}$$

### 20-km Time trial (TT_20_)

As a marker of endurance exercise performance, participants were instructed to complete a 20-km self-paced time trial (TT_20_) as quickly as possible. This test was performed on the participant’s own bike, mounted onto a stationary ergometer (Cyclus2®, Leipzig, Germany). The only feedback given to participants during the trial was distance completed, and participants were allowed to leave the saddle at any time during the time trial. Exercise duration and power were recorded during the trial. Participants were allowed to control the gear ratio throughout the entire time trial, corresponding to three gearings on the chain-ring (28, 39 and 52) and ten on the rear sprocket (23, 21, 19, 17–11). The consumption of water was allowed *ad libitum* during this test.

### High-intensity interval training (HIT)

The HIT protocol used in this study has previously been shown to improve endurance performance in highly-trained cyclists^26^. All participants completed 12 training sessions over a period of 4 weeks. Each HIT session was comprised of 8 exercise intervals, performed at a workload corresponding to baseline P_peak_. The interval duration was set at 60% of baseline T_max_ (mean = 172.8 ± 32.8 s; range = 132.6 to 242.3 s) and there was a 1:2 work to rest ratio. Dependent on compatibility, participants were provided with a power meter (Stages Powermeter Crank System, Boulder, USA, or SRM, Schoberer Rad Meßtechnik, Jülich, Germany**)** and GPS sensor (Garmin Edge 500, Olathe, Kansas, USA) to conduct training independently and on their own bicycle. All training data was uploaded to an online training platform (Garmin Connect, USA), and continually monitored by study investigators to ensure participants were reaching their training targets. If the power meter was not compatible, participants performed their training sessions in the laboratory on a stationary ergometer (Lode, Groningen, The Netherlands). All training sessions were performed in the afternoon between ~1400 and 1700.

### Experimental interventions

Approximately 15 min after completing each training session, participants performed their assigned condition. Participants assigned to the WBC condition walked (~2 min) to the medical department of the National Institute of Sport, Expertise and Performance (INSEP, Paris, France). All WBC sessions were performed under the supervision of a doctor, who had visual and auditory contact with participants at all stages of the protocol. The WBC system was comprised of three contiguous chambers maintained at −10 °C, −60 °C and −110 °C (Zimmer Elektromedizin, GmbH, Ulm, Germany). Before cold exposure, participants were instructed to towel dry themselves of any sweat, and were provided with cotton gloves, socks, shoes, a headband, and a mask to protect their extremities. Participants were also required to wear swimwear and/or shorts, and all jewellery, piercings, glasses, and contact lenses were removed prior to cold exposure. Once adequate preparation was confirmed by the doctor, participants passed through the −10 °C and −60 °C chambers, and remained in the −110 °C chamber for 3 min, as previously described^1^. A similar WBC protocol (4 min at −110 °C) has previously been reported to significantly reduce core (0.3 °C) and muscle (1.6 °C at 3 cm depth, *vastus lateralis* muscle) temperatures from baseline, 1 h after exposure. During cold exposure, participants were instructed to walk slowly around the chamber. Participants in the CON condition were instructed to sit at room temperature (23 °C) for the same duration as the WBC condition (3 min). All WBC sessions were performed in the afternoon between ~1500 and 1800.

### Sleep analysis

Baseline sleep data were recorded for 5 consecutive days before starting the 4-week training intervention. Every night throughout the study, participants wore a wrist actigraph (Actiwatch; CamNTech Inc., England) to monitor sleep patterns and manually documented their sleep in a sleep diary. They were asked to press the time stamp button on the actigraph upon switching the lights off (considered as the bed time) and to press it again upon waking up (sleep end) and to take it off upon getting up. The sleep variables used for analysis were as previously described^9^ and as follows:

- Total time in bed (bed time to get-up time)
- Bed time, sleep start, sleep latency (sleep start - bed time), and get-up time,
- Actual sleep time (assumed sleep time - wake time as determined by the algorithm)
- Sleep efficiency: actual sleep time/total time in bed.

No instructions were given to the participants to alter their normal sleeping patterns. Values obtained nightly within each training period were then averaged for subsequent statistical analysis. Actigraphy is a valid alternative to Polysomnography for measuring the sleep of elite athletes^49^. A high actigraphy sensitivity threshold was selected to detect sleep parameters since it has been shown that the cut-off value of 80 activity counts yield the best combination of sensitivity and specificity in a population of elite athletes^49^. Correlations between Actiwatch and Polysomnography are r = 0.82 to 0.65 for total sleep time and sleep efficiency^50^.

### Blood analyses

Blood samples were collected 5 min before both the baseline and post-training SM_120_. To prevent within-participant circadian variability, pre vs post-training blood samples were collected at the same time of day for the corresponding participant. A 20-gauge hypodermic needle (Grenier Bio-One, Kremsmünster, Austria) was inserted into an antecubital vein before blood draw. Samples (approximately 10 mL each) were collected into both EDTA and SST vacutainers (Greiner Bio-one, Kremsmünster, Austria). Both tubes were centrifuged at 1000 *g* and 4 °C for 15 min, immediately after collection for the EDTA tubes and after sitting at room temperature for 30 min for SST tubes. The resultant plasma (EDTA) and serum (SST) were collected into 1.5 mL aliquots (Eppendorf) and stored at −80 °C for subsequent analyses. Plasma samples were analysed for catecholamine (adrenaline/noradrenaline) concentrations by enzyme-linked immunosorbent assay (ELISA) with commercially-available kits (Demeditec Diagnostics GmbH, Kiel, Germany). Serum samples were analysed for cortisol concentrations, also by ELISA (IBL International GMBH, Hamburg, Germany). All samples were analysed in duplicate with a spectrophotometer (Dynex MRXe, Magellan Biosciences, Chelmsford, MA, USA). Due to unforeseen circumstances with blood collection and/or analysis, complete data sets for the blood markers were available for only nine participants in the control group (*n* = 9). The intra-assay coefficients of variation for the adrenaline, noradrenaline, and cortisol ELISA kits were 7.2 ± 5.4%, 13.6 ± 18.7%, and 6.1 ± 6.1%, respectively. As per manufacturer guidelines, minimum detectable concentrations for adrenaline, noradrenaline, and cortisol were 3.3 pg/mL, 1.3 pg/mL, and 2.46 ng/mL, respectively.

### Statistics

Data are reported as mean ± standard deviation. Comparisons were analysed using a two-way general linear model (ANOVA) with repeated-measures for time, where the within-subject factor was time (Pre vs. Post) and the between-subject factor was condition (CON vs WBC). An additional within-subject comparison was made for SM_120_ data (i.e., RER and carbohydrate/fat oxidation rates) to assess changes during the SM_120_ (effect of ‘duration’). Homoscedasticity was confirmed for all dependent variables using Levene’s test of homogeneity. Normality of distribution was assessed using the Shapiro-Wilks test, and nonparametric data not belonging to a particular distribution (i.e., adrenaline, noradrenaline, and cortisol) were analysed using a Friedman two-way ANOVA. The level of significance for all data was set at *P* < 0.05. The statistical analyses were performed using IBM SPSS Statistics V19 (IBM Corporation, USA). To complement the null-hypothesis statistical testing, effect sizes (ES) were calculated to assess the magnitude of the observed effects. The pooled between-subject standard deviation was used for analysis. For observed time effects, ES were reported individually for each condition. Cohen’s conventions for ES (Cohen’s *d* ± 90% confidence intervals) were used for interpretation, where ES = 0.2, 0.5 and 0.8 are considered small, medium and large, respectively^51^.

## Data Availability

The datasets generated during and/or analysed during the current study are available from the corresponding author upon reasonable request.
